# Different ATM Signaling in Response to Chromium(VI) Metabolism via Ascorbate and Nonascorbate Reduction: Implications for *in Vitro* Models and Toxicogenomics

**DOI:** 10.1289/ehp.1409434

**Published:** 2015-05-15

**Authors:** Michal W. Luczak, Samantha E. Green, Anatoly Zhitkovich

**Affiliations:** Department of Pathology and Laboratory Medicine, Brown University, Providence, Rhode Island, USA

## Abstract

**Background:**

Carcinogenic hexavalent chromium [Cr(VI)] requires cellular reduction to generate DNA damage. Metabolism of Cr(VI) by its principal reducer ascorbate (Asc) lacks a Cr(V) intermediate, which is abundant in reactions with a minor reducing agent, glutathione. Cultured cells are widely used in mechanistic studies of Cr(VI) toxicity; however, they typically contain < 1% of normal Asc levels. Asc deficiency is also expected to diminish protection against reactive oxygen species.

**Objectives:**

We assessed how the presence of Asc in cells affects their stress signaling and survival responses to chromate.

**Methods:**

We investigated the effects of Asc restoration in human lung H460 cells and normal human lung fibroblasts on the activation and functional role of ATM kinase, which controls DNA damage responses involving several hundreds of proteins.

**Results:**

Treatment of standard cultures with Cr(VI) strongly activated ATM, as indicated by its automodification at Ser1981 and by phosphorylation of checkpoint kinase 2 (CHK2) and chromatin/transcription regulator KRAB-associated protein 1 (KAP1). Confirming the importance of activated ATM, its inhibition impaired replication recovery and clonogenic survival. In contrast, fully Asc-restored cells lacked ATM activation by Cr(VI), and ATM silencing produced no significant effects on p53 stabilization, apoptosis, replication recovery, or clonogenic survival. Dose dependence studies found a close correlation between ATM activation and the extent of Cr(VI) reduction by glutathione.

**Conclusions:**

Asc restoration in cultured cells dramatically altered their stress responses to Cr(VI) by preventing activation of the oxidant-sensitive ATM network. We suggest that toxicogenomic and other cell response-based approaches likely underestimate Cr(VI) genotoxicity when standard ATM-activating carcinogens are used as references.

**Citation:**

Luczak MW, Green SE, Zhitkovich A. 2016. Different ATM signaling in response to chromium(VI) metabolism via ascorbate and nonascorbate reduction: implications for *in vitro* models and toxicogenomics. Environ Health Perspect 124:61–66; http://dx.doi.org/10.1289/ehp.1409434

## Introduction

Hexavalent chromium [Cr(VI)] is a well-recognized human carcinogen that is found in the workplaces of several millions of workers worldwide [Agency for Toxic Substances and Disease Registry (ATSDR) 2000]. Environmental exposure to Cr(VI) has raised questions about its potential adverse health effects in the general population (Salnikow and Zhitkovich 2008). Intracellular reduction of Cr(VI) to stable Cr(III) is responsible for the production of DNA-damaging products. Cr(VI) metabolism can generate variable amounts of Cr(V) and Cr(IV) intermediates depending upon the nature of the reducing agent. Ascorbate (Asc) is the key reducer of Cr(VI) in cells *in vivo*, accounting for more than 90% of its metabolism (Standeven and Wetterhahn 1991, 1992; Suzuki and Fukuda 1990). The reduction of Cr(VI) by Asc generates Cr(IV) as the main intermediate under physiological conditions (Stearns and Wetterhahn 1994; Zhang and Lay 1996). Cr(VI) reactions with thiol-based secondary reducing agents yield reactive Cr(V) as the first intermediate (Borges et al. 1991; Bose et al. 1992). The final product of Cr(VI) reduction by all reducing agents is Cr(III), which forms several mutagenic Cr–DNA adducts (Zhitkovich 2005). Human and nonhepatic rodent cells in standard cultures contain either undetectable or low micromolar concentrations of Asc (Reynolds et al. 2007, 2012) in contrast to the 1–3 millimolar amounts of Asc in the main tissues *in vivo* (Bergsten et al. 1990; Kojo 2004). Consequently, metabolism of Cr(VI) in cultured cells is dominated by the most abundant thiol, glutathione (GSH) (Zhitkovich 2005), which yields Cr(V) species that can cause oxidative damage via direct or Fenton-like reactions (Sugden and Stearns 2000; Sugden et al. 2001). Findings in cultured cells commonly guide the design and interpretation of expensive animal studies and are used to determine the mode of action for regulatory purposes. Thus, it is critical to ensure that *in vitro* models adequately recapitulate the main metabolic processes for Cr(VI) in tissues.

The main signaling network activated by oxidants and other DNA breakage–inducing agents is initiated by ATM kinase, which regulates phosphorylation of more than 1,000 proteins, including stress-sensitive transcription factors, and consequently orchestrates changes in all major cellular processes, such as DNA repair, chromatin remodeling, gene expression, cell cycle, cell death, metabolism, and others (Ditch and Paull 2012; Shiloh and Ziv 2013). ATM can also be activated by chromatin injury (Kaidi and Jackson 2013) and by direct oxidation of its cysteines (Guo et al. 2010). Given the broad importance of ATM in the regulation of cellular responses to DNA damage and oxidants, examination of the activation of this kinase by Cr(VI) has the potential to uncover critical signaling and survival mechanisms. Asc-deficient human cells treated with Cr(VI) showed some evidence of activated ATM (Ha et al. 2003; Hill et al. 2008; Wakeman et al. 2004), which has not yet been assessed functionally in isogenic systems nor been verified in cells with physiological concentrations of Asc.

In this work, we investigated the activation of ATM signaling and its significance in Cr(VI)-treated human cells with and without the restoration of physiological concentrations of Asc. We found that Cr(VI) caused robust stimulation of ATM in Asc-deficient cells, which increased their replication recovery and long-term survival. In contrast, all of the tested ATM-dependent responses were absent in Asc-restored cells. Thus, the presence of Asc dramatically altered Cr(VI)-induced cell stress responses, which excluded canonical DNA damage signaling by ATM. Lack of ATM activation under physiological conditions of Cr(VI) metabolism has important implications for the use of *in vitro* models in Cr(VI) research and for the assessment of Cr(VI) genotoxicity by toxicogenomic approaches *in vivo*.

## Methods

*Cells and treatments.* All cell lines were purchased from the American Type Culture Collection. H460 cells were grown in RPMI-1640 medium with 10% serum under 95% air/5% CO_2_. Normal IMR90 fibroblasts proliferate better under physiological oxygen tension, and they were cultured in an atmosphere of 5% O_2_/5% CO_2_ using Dulbecco’s Modified Eagle’s Medium and 10% serum. Cells were treated in complete growth media for 3 hr with K_2_CrO_4_ [Cr(VI)] and for 1 hr with camptothecin (CPT). The ATM inhibitors KU60019 (ATM-i1, 1 μM) and KU55933 (ATM-i2, 5 μM) were added simultaneously with Cr(VI). In the time-course study of checkpoint kinase 2 (CHK2) phosphorylation, H460 cells were treated with Cr(VI) for 3 hr and collected for protein extraction at 0, 2, 4, and 8 hr postexposure. Cells were depleted of GSH by preincubation with 0.1 mM buthionine sulfoximine (BSO) for 24 hr before the addition of Cr(VI) (DeLoughery et al. 2014).

*Asc restoration in cells*. Cells were incubated for 90 min with dehydroascorbic acid, and cellular Asc was quantified using 1,2-diamino-4,5-dimethoxybenzene (Reynolds and Zhitkovich 2007). Cellular volumes were calculated from forward scatter profiles generated by flow cytometry (FACSCalibur, BD Biosciences).

*Small hairpin RNA (shRNA)*. pSUPER-retro–based vectors were used to produce stable knockdowns of ATM and CHK2. Targeting sequences for ATM and CHK2 were 5´-GAT​ACC​AGA​TCC​TTG​GAGA-3´ and 5´-AAT​GTG​TGA​ATG​ACA​ACT​ACT-3´, respectively. Oligonucleotides and the linearized vector were incubated with T4 ligase overnight, followed by transformation of the plasmid products into *E. coli* cells. The vectors were packaged into the viral particles by cotransfection with MoMuLV gag-pol and VSVG-encoding plasmids into 293T cells. Virus-containing media were collected 48 hr after transfection, filtered, and added to H460 cells overnight. Infected cells were selected and continously maintained in the presence of 1.5 μg/mL puromycin.

*Western blotting.* Protein extracts were prepared by boiling cells for 10 min in 2% sodium dodecyl sulfate (SDS) buffer (2% SDS, 50 mM Tris-HCl pH 6.8, 10% glycerol). For proteins smaller than 100 kDa, cell extracts were separated on 10% sodium dodecyl sulfate polyacrylamide gel electrophoresis (SDS-PAGE) gels and then electroblotted onto polyvinylidene fluoride (PVDF) membranes using a semi-dry transfer apparatus (PierceG2 Fast Blotter). To detect ATM and phospho-ATM, proteins were separated on 6% polyacrylamide gels and electro-blotted onto PVDF membranes overnight by a cold wet transfer procedure. Primary antibodies were obtained from Cell Signaling Technology [phospho-Thr68-CHK2, poly (ADP-ribose) polymerase (PARP), cleaved caspase 7, CHK2, phospho-S15-p53], Bethyl (phospho-Ser824-KAP1), BD Biosciences (MSH6), Santa Cruz Biotechnology (ATM, p53), Abcam (phospho-Ser1981-ATM), and Sigma-Aldrich (γ-tubulin).

*Scoring of replicating cells*. IMR90 cells were seeded on human fibronectin–coated coverslips and Asc-preloaded on the following day. After being exposed to Cr(VI) for 3 hr, the cells were allowed to recover for 24 hr before being labeled with 10 μM 5-ethynyl-2′-deoxyuridine (EdU) for 1 hr. ATM-i1 (1 μM KU60019) was present during and after exposure to Cr(VI). The cells were fixed with ice-cold methanol for 10 min, and EdU incorporation was visualized using the DNA Click-iT AlexaFluor 488 Imaging Kit (Molecular Probes). Images of DAPI/EdU-stained slides were analyzed using SpotAdvanced 5.1.23 software (Diagnostic Instruments).

*Cell viability.* Cr(VI) cytotoxicity was monitored by measuring the cells’ metabolic activity using a CellTiter-Glo luminescent cell viability assay kit (Promega). Cells were seeded into 96-well optical bottom plates and allowed to attach overnight before treatment with Cr(VI). Cytotoxicity was assessed at 48 hr post-Cr(VI) exposure. Each viability experiment included 5–6 wells per dose.

*Clonogenic survival.* Cells were seeded onto 60-mm dishes and treated with Cr(VI) for 3 hr on the following day. After growing for 7–8 days, colonies were fixed with methanol and stained with a Giemsa solution. Each clonogenic experiment included 3–4 dishes per dose.

*Uptake of Cr(VI).* Cellular chromium was measured by graphite furnace atomic absorption spectroscopy (GF-AAS) (Messer et al. 2006). After removing Cr(VI)-containing media, cells were washed twice with warm phosphate-buffered saline (PBS) and then detached with a trypsin-EDTA (ethylenediaminetetraacetic acid) solution (Gibco 15400-054). Cells were collected at 800 × *g* for 5 min at 4°C and washed two times with cold PBS. Cellular Cr was extracted with hot nitric acid and quantified by GF-AAS (AAnalyst 600 Atomic Absorption Spectrometer; PerkinElmer).

*Cr(VI) reduction.* The rate of Cr(VI) reduction was measured at 37°C by recording the absorbance at 372 nm every 20–30 sec. Reactions contained 50 mM 3-(*N*-morpholino)propanesulfonic acid (MOPS) (pH 7.0), 100 mM sodium chloride (NaCl), 50 μM Cr(VI), and various reducing agents such as thiols [3 mM GSH + 0.2 mM cysteine (Cys)], Asc (0.2–0.6 mM), and mixtures of thiols with Asc.

*Statistics.* Differences between the groups were evaluated using a two-tailed, unpaired *t*-test. Error bars in the figures are SD values.

## Results

**Figure 1:**
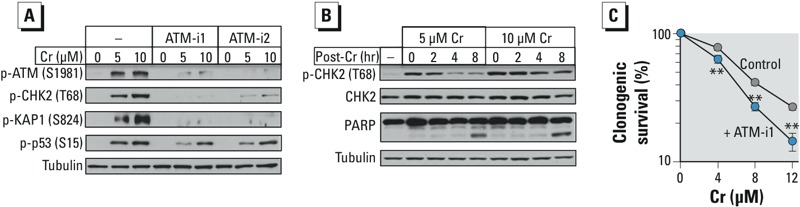
Activation of ATM signaling in H460 cells without Asc restoration. Cells were treated with Cr(VI) for 3 hr in the absence and presence of ATM inhibitors (ATM-i1–1 μM KU60019, ATM-i2–5 μM KU55933). (*A*) ATM-dependent protein phosphorylation immediately after Cr(VI) exposure. (*B*) Western blots of cells at different times after Cr(VI) exposure. (*C*) Clonogenic toxicity of Cr(VI) in the presence and absence of 1 μM ATM-i1. **p < 0.01 relative to cells without inhibitor, *n *= 3.

*ATM activation in standard culture of H460 cells.* We chose H460 human lung epithelial cells as our main cellular model to determine the effects of Cr(VI) on the ATM pathway. These cells display normal DNA damage signaling to a classic ATM activator, ionizing radiation (Zhang et al. 2006), and contain wild-type stress-sensitive transcription factor p53, which showed robust up-regulation in response to genotoxic and nongenotoxic carcinogens (Wong et al. 2012, 2013). Lung is a main target of carcinogenic effects in Cr(VI)-exposed workers (Salnikow and Zhitkovich 2008), making H460 cells a histologically relevant model. Our standard H460 cultures contained 7 ± 4 μM cellular Asc (*n* = 6), which is less than 1% of the normal concentration in the human lung (Slade et al. 1985). To monitor ATM activation, we examined the phosphorylation status of three well-characterized ATM targets: ATM autophosphorylation at Ser1981, CHK2 at Thr68, and transcription/chromatin regulator KAP1 at Ser824 (Shiloh and Ziv 2013). We found that all three proteins showed increased phosphorylation after Cr(VI) exposure (Figure 1A). The addition of two selective ATM inhibitors (KU60019, KU55933) blocked Cr(VI)-induced phosphorylation, confirming its dependence on ATM. A less specific ATM target, Ser15 in the transcription factor p53, also showed greatly increased phosphorylation by Cr(VI), which was partially dependent on ATM as indicated by the suppressive effects of its kinase inhibitors, particularly at the lower Cr(VI) concentration (Figure 1A). A time-course analysis of CHK2 phosphorylation found that ATM activity was highest in cells collected immediately after Cr(VI) exposure, which was followed by a gradual decline over several hours of recovery (Figure 1B). The induction of apoptosis, assayed by PARP cleavage, did not occur until 8 hr post-Cr(VI) exposure, indicating that CHK2 phosphorylation and, by extension, ATM activation were not triggered by apoptotic DNA fragmentation. ATM was important for protection against Cr(VI) toxicity, as indicated by a significant decrease in clonogenic survival of cells in the presence of its kinase inhibitor (Figure 1C).

**Figure 2:**
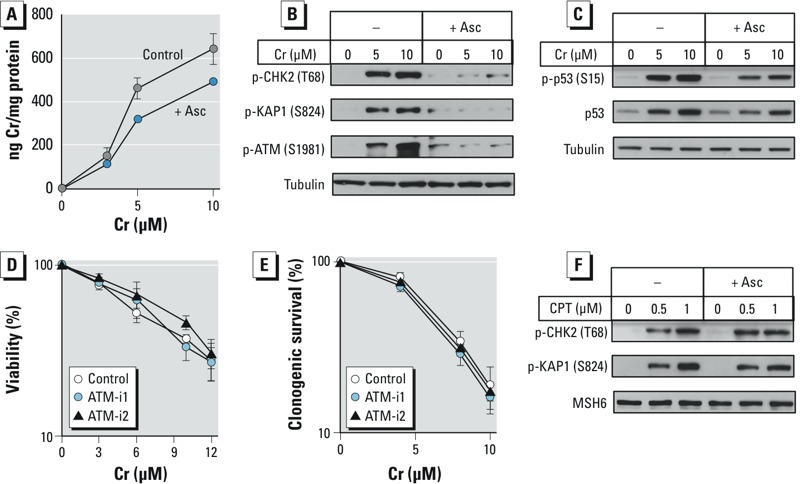
ATM responses in Asc-restored H460 cells. Control and Asc-preloaded cells (+Asc) were treated with Cr(VI) for 3 hr and then immediately collected. Inhibitors: ATM-i1–1 μM KU60019, ATM-i2–5 μM KU55933. (*A*) Cr(VI) uptake (*n *= 3). (*B*) Suppression of ATM activation by Asc restoration. (*C*) Effect of cellular Asc on p53 upregulation by Cr(VI). (*D*) Effect of ATM inhibitors on cell viability (72 hr post–Cr measurements, *n *= 4) and (*E*) clonogenic survival (*n *= 2). Data in panels *D* and *E* are means ± SD. (*F*) Phosphorylation of ATM targets in cells treated with camptothecin (CPT).

*Effects of Asc restoration on ATM activation*. To examine the effects of Asc on ATM, we increased its levels in H460 cells to 2.8 ± 0.3 mM (*n* = 3). Asc-restored (+Asc) cells had, on average, 1.37 times lower accumulation of Cr(VI) (*p* < 0.001) for the three tested concentrations (Figure 2A). The differences in uptake were also statistically significant for individually analyzed 5-μM (*p* < 0.01) and 10-μM (*p* = 0.02) Cr(VI) concentrations. The observed decrease in intracellular Cr(VI) could have been caused by its extracellular reduction to Cr(III), to which the cells were impermeable, by leaked cellular Asc. Mock-preloaded cells showed expected increases in the phosphorylation of all three ATM targets by Cr(VI) but, in striking contrast, no responses were detected in +Asc cells (Figure 2B). This inhibitory effect of cellular Asc on ATM signaling was clearly much more dramatic than its modest decrease of Cr(VI) uptake. +Asc cells also displayed substantially less stress signaling targeting p53, as indicated by its protein and Ser15 phosphorylation levels (Figure 2C). The remaining increases of p53 readouts in +Asc cells likely reflected the contribution of ATM-independent signaling, as evident from the inability of ATM inhibitors to completely suppress p53-Ser15 phosphorylation in Asc-deficient cells (Figure 1A). Consistent with the lack of significant ATM activation, the addition of ATM inhibitors had no effect on the cytotoxicity and clonogenic lethality of Cr(VI) in +Asc cells (Figure 2D,E). Unlike the results observed with Cr(VI), phosphorylation of ATM targets CHK2-Thr68 and KAP1-S821 by the topoisomerase I poison camptothecin was not altered by Asc restoration (Figure 2F), indicating that Asc did not act as a general suppressor of ATM signaling.

**Figure 3:**
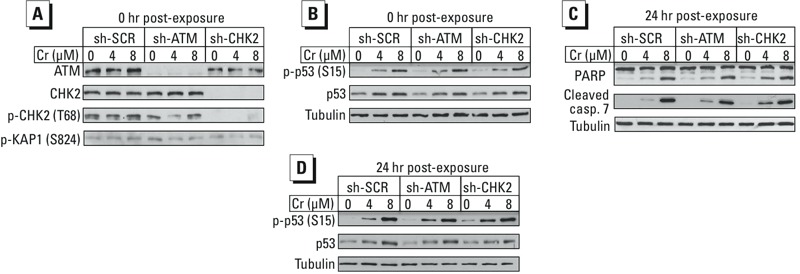
Effects of ATM and CHK2 knockdowns on p53 and apoptotic responses in Asc-restored H460. (*A*) ATM-related phosphorylation in cells collected immediately after 3-hr Cr(VI) exposure. (*B*) Activation of p53 in cells immediately after Cr(VI) exposure. (*C*,*D*) Western blots of cells collected at 24 hr post–Cr exposure. SCR, scrambled.

Next, we employed genetic approaches to further explore the potential involvement of ATM signaling in cytotoxic responses to Cr(VI) in +Asc cells. We constructed stable knockdowns of ATM and its main transducer kinase CHK2 and tested their effects on the activation of the transcription factor p53 and apoptotic responses. Similarly to parental H460 cells with restored Asc, the shRNA-expressing lines showed no increases in phosphorylation of the ATM targets CHK2 and KAP1 after Cr(VI) exposure (Figure 3A). Consistent with the inactivity of the ATM pathway, up-regulation of p53 protein and the phosphorylation of p53 at Ser15 by Cr(VI) were unaffected by ATM or CHK2 depletion (Figure 3B). In cells collected 24 hr after exposure, Cr(VI)-treated samples showed activation of apoptosis as indicated by PARP cleavage and by the formation of cleaved (active) caspase-7. ATM knockdown produced no effects on the apoptotic responses or activation of p53 in these samples (Figure 3C,D). CHK2-depleted cells had slightly higher amounts of cleaved caspase-7 for 4-μM but not 8-μM Cr(VI), although this kinase usually plays a proapoptotic role in ATM signaling (Stracker et al. 2009). It is possible that the loss of CHK2 altered activation of the related kinase CHK1, which can respond to Cr–DNA adducts. Taken together, these results indicate that ATM does not play a significant role in the apoptotic effects of Cr(VI) in H460 cells containing physiological levels of Asc.

**Figure 4:**
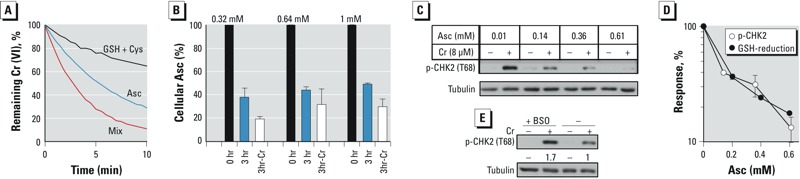
Dose-dependent effects of Asc on CHK2 phosphorylation and Cr(VI) reduction. (*A*) Cr(VI) reduction *in vitro* by 0.3 mM Asc, 3 mM GSH + 0.2 mM Cys, and a mixture of all three reducing agents. (*B*) Loss of Asc from H460 cells during 3-hr incubation with and without 8 μM Cr(VI). Cells were preloaded with 0.32, 0.64, and 1 mM Asc (0 hr) and analyzed for Asc after 3-hr incubation without (3 hr) and with Cr(VI) (3hr-Cr). (*C*) CHK2 phosphorylation in H460 cells preloaded with different concentrations of Asc prior to Cr(VI) exposure for 3 hr. Calculated mid-exposure Asc concentrations are shown. (*D*) Comparison between the amounts of Cr(VI)-induced phospho-CHK2 and the contribution of thiols to Cr(VI) reduction at different Asc levels. Cells were preloaded with Asc and treated with Cr(VI) as in panel C. Data are means ± SD for three independent experiments. (*E*) Effect of GSH depletion on CHK2 phosphorylation. H460 cells were pretreated with 0.1 mM buthionine sulfoximine (BSO) for 24 hr and then exposed to 8 μM Cr(VI) for 3 hr.

*Dose-dependent effects of Asc.* To better understand how Asc suppresses ATM activation, we examined dose-dependent effects of Asc in cells and analyzed its rates of Cr(VI) reduction. The small thiols GSH and Cys are responsible for Asc-independent reduction of Cr(VI) in cells (Zhitkovich 2005). We determined previously that H460 cells contain approximately 3 mM GSH (Reynolds et al. 2012) and 0.2 mM Cys (DeLoughery et al. 2014). Figure 4A shows that the rate of Cr(VI) reduction by 0.3 mM Asc was 2.4 times faster than that by a 10-fold higher concentration of cellular thiols (3 mM GSH + 0.2 mM Cys). Despite the clear kinetic superiority of Asc, mixing it with thiols yielded an approximately additive increase in Cr(VI) reduction, indicating a continuing independent contribution of GSH/Cys. Based on a series of additional kinetic studies, we calculated the contribution of the physiological mixture of thiols (3 mM GSH + 0.2 mM Cys) in the presence of different concentrations of Asc. To determine whether the extent of Cr(VI) metabolism by thiols is correlated with the degree of ATM activation, we next examined the induction of CHK2 phosphorylation by Cr(VI) in H460 cells preloaded with different Asc concentrations. Because Asc leaks from cells in culture (Reynolds and Zhitkovich 2007), we first measured the amount of Asc in H460 cells at the end of our standard 3-hr-long incubations. We found that cells lost approximately 50–60% of their Asc without Cr(VI) and 70–80% in the presence of 8 μM Cr(VI) (Figure 4B). There was a statistically borderline trend for a larger depletion of Asc by Cr(VI) in the cells preloaded with the lowest dose (*p* = 0.067 for 0.32-mM samples + Cr vs. 1-mM samples + Cr). The loss of Asc in culture is not limited to established cell lines; freshly purified human lymphocytes also lost their Asc during *in vitro* incubation (Bergsten et al. 1990). It is currently unclear whether cells lose Asc through passive or active mechanisms, nor is it clear which factors regulate this process. It is possible that the absence of Asc in the extracellular medium triggers active efflux of cellular Asc to establish antioxidant defense of the outer layer of the plasma membrane. Our attempts to maintain cellular Asc levels using the continuous presence of reduced and/or oxidized Asc have been unsuccessful to this point, possibly due to oxidation of Asc in the iron-rich culture media. Using the initial and final concentrations of cellular Asc, we calculated its mid-exposure levels and included them on the *x*-axis in dose-dependent analyses of phospho-CHK2 induction by Cr(VI) (Figure 4C). The amounts of phospho-CHK2 and the percentage of Cr(VI) reduction by thiols showed similar inverse dose dependence on Asc (Figure 4D), suggesting that a shift to the thiol-independent Cr(VI) metabolism was a likely cause of the ATM-suppressive effects of cellular Asc. A unique feature of Cr(VI) reduction by GSH and Cys is the direct production of Cr(V) intermediate via the initial one-electron transfer reaction (Zhitkovich 2005). Depletion of GSH increases the abundance of Cr(V) owing to its extended stability under low concentrations of cellular reducers (DeLoughery et al. 2014). The amount of phospho-CHK2 by Cr(VI) was also increased in GSH-depleted cells (Figure 4E), which is consistent with the ability of Cr(V) to promote ATM activation.

**Figure 5:**
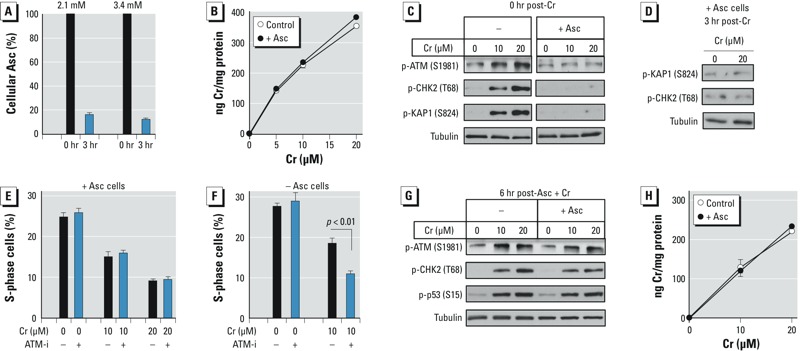
ATM activation in IMR90 normal human lung cells. Control and Asc-restored (+Asc, 3.4 mM Asc) cells were treated with Cr(VI) for 3 hr. Bar and line graphs show means ± SD, *n *= 3. (*A*) Asc levels in cells before and after 3-hr incubation in regular medium. (*B*) Uptake of Cr(VI) by control and Asc-restored (+Asc, 3.4 mM Asc) cells. (*C*) Western blots of cells collected immediately after Cr(VI) exposure. Images of control and +Asc samples are from the same blots, which were cropped to remove unrelated intervening lanes. (*D*) Western blots of +Asc cells collected 3 hr after exposure to Cr(VI). (*E*) Percentage of +Asc cells in S-phase at 24 hr post-exposure. ATM-i (1 μM KU60019) was present during Cr(VI) exposure and subsequent 24-hr incubations. (*F*) As in panel E, except that cells were treated with Cr(VI) without Asc restoration. (*G*) ATM activation and p53 phosphorylation in cells treated with Cr(VI) at 6 hr after Asc loading (3-hr Cr exposure, immediate collection). (*H*) Cr(VI) uptake by cells treated at 6 hr post–Asc loading. In panels G and H, +Asc cells contained 0.110 ± 0.006 mM Asc at the start of Cr exposure.

*ATM responses in normal human lung cells*. We used IMR90 lung fibroblasts to test the effects of cellular Asc in normal human cells. These cells showed large losses of Asc after 3-hr-long incubations, retaining only approximately 15% of the initially loaded amounts (Figure 5A). In subsequent experiments, we preloaded IMR90 cells with an initial concentration of 3.4 mM Asc, which at the midpoint of our standard 3-hr-long exposures dropped to 1.3 mM. Uptake of Cr(VI) by IMR90 cells was unaffected by preloading with Asc (Figure 5B). Similarly to H460 cells, Cr(VI) induced robust phosphorylation of ATM targets in control IMR90 cells (12 ± 6 μM cellular Asc), but these responses were prevented by Asc restoration (Figure 5C,D). The ability of Cr(VI)-treated +Asc IMR90 cells to continue replicating, as indicated by the percentage of S-phase cells at 24 hr post-exposure, was not altered by the inhibition of ATM by KU60019 (Figure 5E), further supporting the lack of a biologically significant activation of this kinase. In accord with the western blotting results showing the activation of ATM in Asc-deficient IMR90, addition of the same ATM inhibitor to these cells increased the replication-inhibitory effects of Cr(VI) (Figure 5F). To test whether Asc restoration changes cellular physiology, we examined stress signaling in IMR90 cells treated with Cr(VI) at 6 hr post–Asc loading. At this time point, the cells retained only 3.2 ± 0.2% of the initially loaded Asc (3.4 mM Asc in freshly loaded cells). We found that a loss of cellular Asc resulted in the reestablishment of ATM activation and p53 phosphorylation by Cr(VI) without any changes in metal accumulation (Figure 5G,H). Thus, suppression of ATM signaling by Asc required the presence of Asc during Cr(VI) exposure, which again points to Cr(VI) metabolism as the primary target of Asc-induced effects.

## Discussion

*Effects of reducers on ATM activation by Cr(VI).* Our results showed that Cr(VI) was capable of robust activation of the apical DNA damage–responsive kinase ATM in normal and transformed human lung cells grown under standard Asc-deficient conditions. The inhibition of ATM activity in these cells decreased their clonogenic survival and replication recovery following Cr(VI) exposure, demonstrating the toxicological importance of ATM signaling. In a striking contrast, we found no biochemical or functional evidence for ATM activation by Cr(VI) in Asc-restored cells. ATM activity toward its major target CHK2 was already decreased by 50% at an approximately 1:20 ratio of cellular Asc to GSH, and it was completely suppressed at a 1:4 ratio. *In vivo* cellular Asc and GSH are present in ratios ranging from 0.5:1 to 2:1 (Kojo 2004; Meister 1994). In the absence of Asc, Cr(VI) is reduced in cells by thiols (primarily by GSH) via one-electron transfer reactions, resulting in the production of reactive Cr(V) (Zhitkovich 2005). Asc initiates Cr(VI) reduction via two-electron transfer, yielding Cr(IV) as the sole intermediate (Stearns and Wetterhahn 1994; Zhang and Lay 1996). Studies using redox-sensitive probes *in vitro* and in cells showed that Cr(V) but not Cr(IV) species can act as oxidants (DeLoughery et al. 2014). Taken together with the observed close correlation of thiol-mediated reduction and CHK2 phosphorylation (Figure 4D), this evidence suggests that Cr(V) intermediates were likely responsible for ATM activation by Cr(VI) in Asc-deficient cells. The absence of ATM activation in +Asc cells is consistent with the suppression of oxidative DNA damage by Cr(VI) in Asc-replete cells (Reynolds et al. 2012). However, cellular Asc does not diminish all forms of Cr(VI) genotoxicity because uptake-normalized formation of Cr–DNA adducts, DNA–protein crosslinks or DNA and chromosomal breaks have been found to be either similar or even higher in Asc-restored cells (Macfie et al. 2010; Reynolds and Zhitkovich 2007; Reynolds et al. 2007, 2009).

Oxidants can trigger ATM activity as a result of either the production of DNA double-strand breaks (Shiloh and Ziv 2013) or ATM dimerization via oxidation of its cysteines (Guo et al. 2010). A characteristic feature of ATM activated by cysteine oxidation is the lack of phosphorylation of chromatin-bound targets, such as KAP1. We observed phosphorylation of both KAP1 and a soluble protein, CHK2, suggesting that DNA damage was involved in stimulating ATM activity. Genetic approaches revealed significant formation of oxidative DNA damage by Cr(VI) in Asc-deficient but not in Asc-restored cells (Reynolds et al. 2012). Thus, it is likely that oxidative DNA damage played a major role in ATM activation during non-Asc reduction of Cr(VI). Cr(VI) metabolism also causes oxidation of protein thiols (Myers 2012), and the direct oxidation of ATM cysteines triggering kinase activity cannot be ruled out. In addition to the formation of reactive Cr(V) during one-electron reduction of Cr(VI), oxidative stress in Asc-deficient cells is further promoted by the generation of GS^•^ radicals, the elimination of which is associated with the formation of superoxide and H_2_O_2_. Asc is a preferred cellular scavenger for various reactive oxygen species, and its radical does not form toxic byproducts (Winterbourn 2008).

*Toxicological implications*. ATM is a major coordinator of DNA damage responses, and directly or via its downstream kinases and other transducers, it controls the phosphorylation status of more than 1,000 proteins in cells exposed to oxidants (Ditch and Paull 2012; Shiloh and Ziv 2013). The ATM-dependent signaling network regulates approximately 350 proteins involved in gene-expression control, including several major transcription factors such as p53, activating transcription factor 1 (ATF1), E2F transcription factor 1 (E2F1), and nuclear factor kappa-light-chain-enhancer of activated B cells (NF-κB). A broad spectrum of protein targets and cellular functions controlled by ATM makes this kinase a dominant regulator of stress responses to many DNA-damaging agents. The inability of Cr(VI) to activate ATM in Asc-restored cells indicates that despite clear evidence of its genotoxicity, such as activation of nucleotide excision repair, Cr–DNA adduction, and DNA and chromosomal breakage even at low exposure levels (DeLoughery et al. 2015; Reynolds et al. 2007, 2009; Zhitkovich 2011), Cr(VI)-induced signaling is expected to lack many typical features of DNA damage responses. Supporting this suggestion are the findings that Cr(VI)-induced apoptosis in Asc-restored cells was p53-independent (Reynolds and Zhitkovich 2007), and histone H2AX phosphorylation by Cr(VI)-induced DNA breaks was mediated by ATR, not ATM kinase (DeLoughery et al. 2015). DNA double-strand breaks by Cr(VI) are selectively formed in the euchromatin of Asc-restored cells (DeLoughery et al. 2015). Although ATM is typically a central regulator of cellular responses to DNA double-strand breaks, it is dispensable for repair of euchromatic breaks (Goodarzi et al. 2008; Rass et al. 2013), explaining the observed lack of functional importance of ATM for recovery after Cr(VI) exposure. Stress signaling by DNA–protein crosslinks, which is another form of Cr–DNA damage, is also dependent on ATR kinase, not ATM (Wong et al. 2012).

Classification of Cr(VI) as a genotoxic or nongenotoxic carcinogen has a major impact on its risk assessment, calling for the use of linear or nonlinear low-dose extrapolation models, respectively (Zhitkovich 2011). One of the current approaches to understanding mechanisms of toxicity or modes of carcinogenic action relies on the characterization of perturbed cellular pathways, which is most commonly done by examining gene expression profiles. Transcriptome analyses of the small intestines of mice exposed to Cr(VI) in drinking water did not detect a classic genotoxic signature, which was taken as evidence of a nongenotoxic mode of action in this tissue (Kopec et al. 2012; Thompson et al. 2012). Alternatively, the inability of these studies to detect typical DNA damage–associated transcriptome changes can be explained by our findings of an absence of ATM activation during Cr(VI) metabolism by its principal physiological reducer, Asc.

## Conclusions

The near absence of Asc in cells maintained under standard culture conditions resulted in distorted stress responses to Cr(VI) due to its metabolism by secondary reducers. Cr–DNA damage in cells with restored Asc did not engage a classic genotoxic signaling pathway relying on ATM kinase. We suggest that the assessment of Cr(VI) genotoxicity *in vivo* by transcriptome profiling and other indirect analyses should include reference carcinogens with ATM-independent DNA damage responses in the tested tissues.
